# Essential oil of *Pinus koraiensis* inhibits cell proliferation and migration via inhibition of p21-activated kinase 1 pathway in HCT116 colorectal cancer cells

**DOI:** 10.1186/1472-6882-14-275

**Published:** 2014-07-30

**Authors:** Sun-Mi Cho, Eun-Ok Lee, Sung-Hoon Kim, Hyo-Jeong Lee

**Affiliations:** Cancer Preventive Material Development Research Center, College of Korean Medicine, Kyung Hee University, 1 Hoegi-dong, Dongdaemun-gu, 130-701 Seoul, Republic of Korea

**Keywords:** EOPK, PAK1, Colorectal cancer, Proliferation, Migration

## Abstract

**Background:**

The essential oil of *Pinus koraiensis* (EOPK) is biologically active compound obtained from the leaves of *P. koraiensis*. The goal of this study was to investigate the anti-cancer mechanism of EOPK in HCT116 colorectal cancer cells.

**Methods:**

HCT116 cell proliferation was assessed by conducting crystal violet and BrdU assays. To assess the effects of EOPK on cell migration, we performed a wound-healing assay. Further, the contribution of PAK1 to EOPK-induced AKT and extracellular signal-regulated kinase (ERK) suppression was assessed by siRNA-mediated *PAK1* knockdown. Changes to the expression and phosphorylation of PAK1 and its effectors were determined by western blotting, and changes to the actin cytoskeleton were determined by performing an immunofluorescence assay.

**Results:**

EOPK significantly decreased HCT116 cell proliferation and migration, and induced G1 arrest without affecting normal cells. Additionally, EOPK suppressed the expression of PAK1, and decreased ERK and AKT phosphorylation in HCT116 cells. Finally, EOPK suppressed β-catenin, cyclin D1, and CDK4/6 expression.

**Conclusions:**

Our studies indicate that EOPK significantly reduced proliferation and migration of colorectal cancer cells. Furthermore, EOPK suppressed PAK1 expression in a dose-dependent manner, and this suppression of PAK1 led to inhibition of ERK, AKT, and β-catenin activities. Our findings suggest that EOPK exerts its anticancer activity via the inhibition of PAK1 expression, suggesting it may be a potent chemotherapeutic agent for colorectal cancer.

## Background

Colorectal cancer is one of the most common forms of cancer in the Western world [1]. The pathogenesis of colorectal cancer involves several processes, including enhanced cell survival, differentiation, and proliferation [2]. Importantly, K-Ras and B-Raf mutations have been identified in 40% and 10% of colorectal cancers, respectively [3, 4]. Mutations in K-Ras and B-Raf activate the phosphoinositide 3-kinase (PI3K)/AKT and mitogen-activated protein kinase (MAPK)/extracellular signal-regulated kinase (ERK) signaling pathways. In colorectal cancer, overexpression of PI3K/AKT and MAPK/ERK promotes cell survival, proliferation, and migration [5, 6].

The p21 activated kinase (PAK) family of proteins has been identified as a novel target for cancer therapies [7]. PAKs are downstream effectors of the Rho family GTPases, which regulate cell motility and survival. There are six isoforms of PAK that are classified into two families, Group I (PAK1-3) and Group II (PAK4-6). Group I PAKs differ from Group II PAKs in both structure and mechanisms of activation [8]. PAK1 is an important effector of Rac and Cdc42 that regulates cell transformation and tumor proliferation [9]. Activated PAK1 enhances cell survival and migration via the AKT pathway, and stimulates transformation through the Ras/Raf/ERK/MAPK pathway. PAK1 knockdown was shown to inhibit the activation of β-catenin in colorectal cancer, thereby inhibiting Wnt/β-catenin signaling and proliferation [10]. In addition, PAK1 overexpression occurs in a variety of human cancers, including breast, ovarian, and colorectal cancer. Thus, PAK1 may play an important role in cell growth, adhesion, migration, and survival in colorectal cancer, through the activation of AKT, ERK and β-catenin [11–14].

*Pinus koraiensis*, generally called the Korean nut pine, is an evergreen tree found in Korea, China, Japan, and eastern Russia. Unlike the common Pinaceae plants, *P. koraiensis* shows leaves (needles) in fascicles (bundles) of five. Essential oil derived from *P. koraiensis* (EOPK) contains a number of components, including D-limonene, β-pinene, 4-carene, camphene, β-phellandrene, and fencyl [15]. Much is known about the role of EOPK in obesity. For example, our group previously reported that EOPK has an >anti-hyperlipidemic effect through the up-regulation of the low-density lipoprotein receptor and the inhibition of acyl-coenzyme A [15]. Further, a recent report on the effects of EOPK indicated that the oil has anti-obesity and hypolipidemic activity *in vitro* and *in vivo*, using 3 T3-L1 cells and high fat diet-fed rats [16]. Additional studies have shown that EOPK is a potential anti-diabetic agents and that the nut of *P. koraiensis* has antioxidant activity *in vivo* [17, 18]. Others studies have demonstrated that the Korean pine nut is an appetite suppressant, and reduced weight gain in mice fed a high fat diet [19, 20]. Nevertheless, it remains unclear whether EOPK may have an anti-cancer effect. In the present study, we examined the antitumor mechanism of EOPK *in vitro* in HCT116 colorectal cancer cells.

## Methods

### Preparation of essential oil from *P. koraiensis*leaves

Leaves were harvested from a Korean pine tree that was more than 30 years old at the end of October in Gangwondo Honchengun where is famous for producing excellent Korean pine nuts in Korea. The leaves were purchased as a dried condition from agricultural corporation, Beaksongyounglim (Ganwondo, Korea) and identified by the department of Oriental medicine Biotechnology at the Kyung Hee University. A voucher specimen (no.KMH-0081025) was maintained at the herbarium of the department of plant physiology at the Kyung Hee University. EOPK was prepared using a hydrodistillation method [21]. Dried and pulverized *P. koraiensis* leaves were immersed in distilled water and steam distilled using an apparatus with a condenser (Hanil Labtech, Seoul, Korea) for 3 to 4 h at 90°C. The volatile compounds were contained in the water-soluble fraction, and were allowed to settle for 20 min. The essential oil layer was separated and purified by microfiltration.

### Cell culture

Colon26L5, a murine colorectal cancer cell line; NIH-3 T3, a fibroblast cell line; HCT116, a human colorectal cancer cell line; and HCT15, HT29, and SW620, three human colorectal adenocarcinoma cell lines, were purchased from American Type Culture Collection (ATCC) (Rockville, MD), and maintained in RPMI1640 medium supplemented with 10% fetal bovine serum (FBS), 2 μM l-glutamine, and penicillin/streptomycin (WelGene, Deagu, South Korea) in a humidified atmosphere of 5% CO_2_ at 37°C.

### Cytotoxicity assay

Cytotoxicity of EOPK was evaluated by 3-(4,5-dimethylthiazol-2-yl)-2,5-diphenyl tetrazolium bromide (MTT) (Sigma Aldrich, St Louis, MO) assay. The cell were seeded at density of 2 × 10^4^ cells per well in a 96 well plate, cultured for 24 h, and then treated with various concentrations of EOPK (0, 25, 50, 100 μg/ml). After 24 h incubation, Per 50 μl of MTT solution (1 mg/ml) was add to each well and incubated for 2 h at 37°C in dark. The viable cell number was correlated with the production of formazan, which was dissolved with dimethyl sulfoxide (DMSO) and optical density (O.D.) was measured by microplate reader (Molecular Devices Co., Sunnyvale, CA) at 570 nm. Cell viability was calculated by the following equation. Cell viability(%) = [O.D.(EOPK)-O.D.(blank)]/[O.D(control)-O.D.(blank)] × 100.

### Western blot analysis

Cells were lysed in RIPA buffer (50 mM Tris–HCl, pH 7.4, 150 mM NaCl, 1% NP-40, 0.25% sodium deoxycholate, 1 M EDTA, 1 mM Na_3_VO_4_, 1 mM NaF, and protease inhibitors cocktail). Protein samples were quantified using the Bio-Rad DC protein assay kit II (Bio-Rad, Hercules, CA), separated by electrophoresis on an 8 to 10% SDS-PAGE gel, and transferred onto a Hybond ECL transfer membrane (Amersham Pharmacia, Piscataway, NJ). The membranes were blocked in 3% nonfat skim milk and probed with primary antibodies for PAK1 (Abcam, Cambridge, UK), PI3K (Millipore, Billerica, MA, USA), phospho-ERK, ERK, β-catenin (Cell Signaling, Beverly, MA), phospho-AKT, AKT, PTEN (Santa Cruz Biotechnologies, Santa Cruz, CA), or β-actin (Sigma Aldrich Co., St. Louis, MO). Membranes were exposed to horseradish peroxidase (HRP)-conjugated anti-mouse or rabbit secondary antibodies. Protein expression was examined by using an enhanced chemiluminescence (ECL) system (Amersham Pharmacia, Piscataway, NJ).

### siRNA transfection

The PAK1 small interfering RNA (siRNA) I and II were purchased from Cell Signaling. A control siRNA were purchased from Santa Cruz Biotechnology. To transfect the siRNA, HCT116 cells were plated at a density of 1 × 10^5^ cells per well in a six-well plate. Cells were transfected using 100 nM of PAK1 siRNA with siRNA transfection reagent for 48 h. After treatment, cells were stimulated for Western blot or immunofluorescence assay.

### Wound healing assay

The ability of cells to migrate was assayed by wound healing assay. The HCT116 cells (1 × 10^6^ cells/ml) were seeded in a 6-well plate and incubated at 37°C. When confluent, the cells were scratched with a 200-μL pipette tip, followed by washing with PBS. The cells were then treated with EOPK in complete medium for 24 h. After incubation, the cells were fixed and stained with Diff-Quick. Randomly chosen fields were photographed under a fluorescence microscope (AXIO observer A1, ZEISS, Germany). The number of cells that migrated into the scratched area was calculated.

### Cell growth assay

The cell growth assay was performed to measure the anti-proliferative effect of EOPK. HCT116 cells (1 × 10^5^ cells/ml) were seeded in a 6-well plate and incubated at 37°C for 24 h, followed by treatment with various concentrations of EOPK (0, 25, 50, 100 μg/ml). Cells were incubated for 5 d, with daily addition of fresh media and EOPK. To assess proliferation, a crystal violet assay was performed. The medium was removed carefully by mild suction, and 2 ml of 1% glutaraldehyde solution (JUNSEl, Tokyo, Japan) in PBS was added to each well for 15 min at 37°C. After washing with PBS, 2 ml of 0.05% crystal violet (Sigma Aldrich, St Louis, MO) was added for 30 min. Following incubation, the crystal violet solution was removed, and the cells were washed gently with DI water. The plates were then dried room temperature overnight. The following day, a 70% ethanol solution was added (2 ml/well) to each well of the 6-well plate. To release the crystal violet, the plates were placed on a rotary shaker for 2 h at room temperature. The optical density (OD) was measured using a microplate reader (Molecular Devices Co., Sunnyvale, CA) at 570 nm, with a reference filter at 405 nm.

### Proliferation assay

Cell proliferation in HCT116 cells following EOPK treatment was evaluated using a cell proliferation ELISA kit (Roche, Swiss) according to the manufacturer’s instruction. Briefly, after a 24- or 48-h treatment with EOPK, the cells were treated with bromodeoxyuridine (BrdU, 10 μL/well), and incubated for 4 h at 37°C. Following incubation, the BrdU solution was removed, and 200 μl of FixDenat was added to each well. After incubation for 30 min at room temperature, the FixDenat solution was removed and 100 μl of anti-BrdU-POD was added to each well. After washing with PBS three times, 100 μl of substrate solution was added to each well, and the optical density was measured at 450 nm using a microplate reader (Molecular Devices Co., Sunnyvale, CA, USA). All samples were prepared in triplicate, and the assay was repeated at least three times.

### Cell cycle assay

HCT116 cells were treated with EOPK (100 μg/ml) for 24 h. The cells were washed and fixed in 70% ethanol overnight at -20°C. The following day, the cells were treated with RNase A (10 mg/ml) for 1 h at 37°C. The cells were stained by adding 1 ml of propidium iodide (PI) (50 μg/ml). After filtering with nylon mesh (40 μm), the DNA contents of stained cells were analyzed using Cellquest Software (BD Biosciences, San Jose, CA) with a FACSCalibur flow cytometer (Becton Dickinson, Franklin Lakes, NJ).

### Immunofluorescence assay

Cells were plated onto poly-L-lysine coated coverslips and fixed with 4% (v/v) methanol-free formaldehyde solution (pH 7.4) at 4°C for 25 min. The cells were permeabilized with 0.2% Triton-X 100 in PBS for 5 min, and blocked in blocking buffer (5% bovine serum albumin, 0.5% Tween-20 in PBS) for 2 h. The cells were incubated with 200 μl of 100 nM rhodamine phalloidin (Cytoskeleton, USA) at room temperature in the dark for 30 min. The DNA was counterstained for 30 s with 300 μl of 100 nM DAPI in PBS. The slides were mounted with mounting medium (Vector Laboratories, Inc., Burlingame, CA) and visualized using the DV Elite™ Imaging System (Deltavision, Applied Precision, Inc., USA).

### Data analyses

All data were shown as mean ± SE. Statistically significant differences were evaluated using Student’s *t*-test and a Tukey-Kramer multiple-comparison post-test.

## Results

### PAK1 mediates the MEK/ERK, PI3K/AKT, and Wnt/β-catenin signaling pathways in colorectal cancer cells

**Figure 1:**
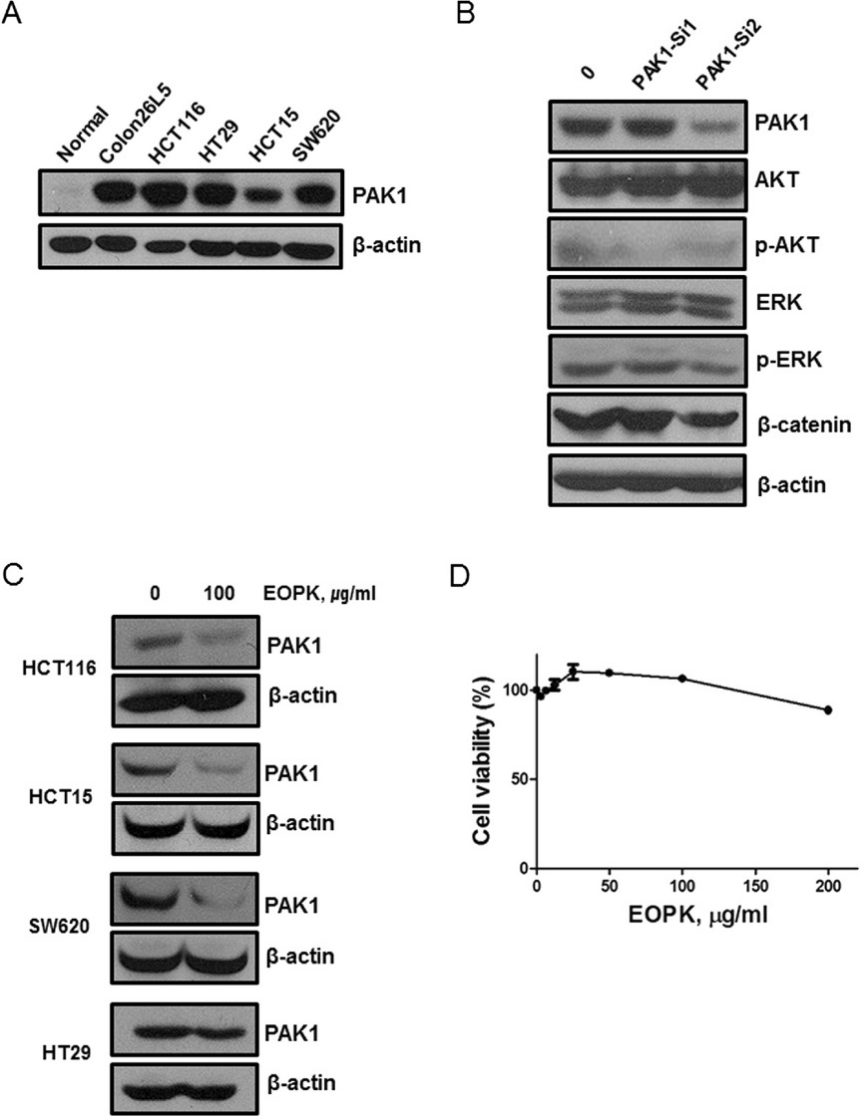
**PAK1 mediates MEK/ERK, PI3K/AKT, and Wnt/β-catenin in colon cancer cells. (A)** Basal levels of PAK1 expression in colorectal cancer cell lines (human and murine) and normal colon cells extracted from mouse tissue were determined by Western blotting. **(B)** HCT116 cells were transfected with PAK1-siRNA1, PAK1-siRNA2, or control siRNA for 48 h. Cell lysates were prepared and subjected to Western blotting to determine the expression of PAK1, p-PAK1, AKT, p-AKT, ERK, p-ERK, β-catenin and β-actin. **(C)** Various human colorectal cancer cells were treated with EOPK (100 μg/ml) for 24 h. **(D)** Cytotoxicity of EOPK was analyzed by MTT assay in NIH-3 T3 cells as a normal cell control.

PAK1 expression increases with the progression of colorectal cancer [6]. Therefore, we first assessed PAK1 expression levels in mouse and human colorectal cancer cell lines by Western blot. PAK1 was expressed in colorectal cancer cell lines including Colon26L5, HCT116, HCT15, HT29, and SW620 cells. PAK1 expression was significantly lower in normal murine colon cells than in cancer cells (Figure 1A).

PAK1 is a central node for various oncogenic signaling pathways, including the Ras/Raf/MAPK/ERK and the PI3K/AKT signaling pathways [8, 22, 23]. To assess the contribution of PAK1 to oncogenic signaling pathways in colon cancer cells, we knocked down PAK1 expression with siRNA. siRNA-mediated PAK1 knockdown attenuated both β-catenin expression and the phosphorylation of AKT and ERK (Figure 1B).

### EOPK inhibits the expression of PAK1

To investigate the effect of EOPK on PAK1, the cells were treated with EOPK (100 μg/ml) and Western blotting was performed. EOPK significantly suppressed the protein expression of PAK1 in colon cancer cell lines, as shown in Figure 1C. Further, we assessed the cytotoxicity of EOPK in NIH-3 T3 cells as a normal cell control. EOPK had no significant cytotoxic effect in NIH-3 T3 cells (Figure 1D).

### EOPK induces G1-arrest and inhibits cell proliferation and migration

**Figure 2:**
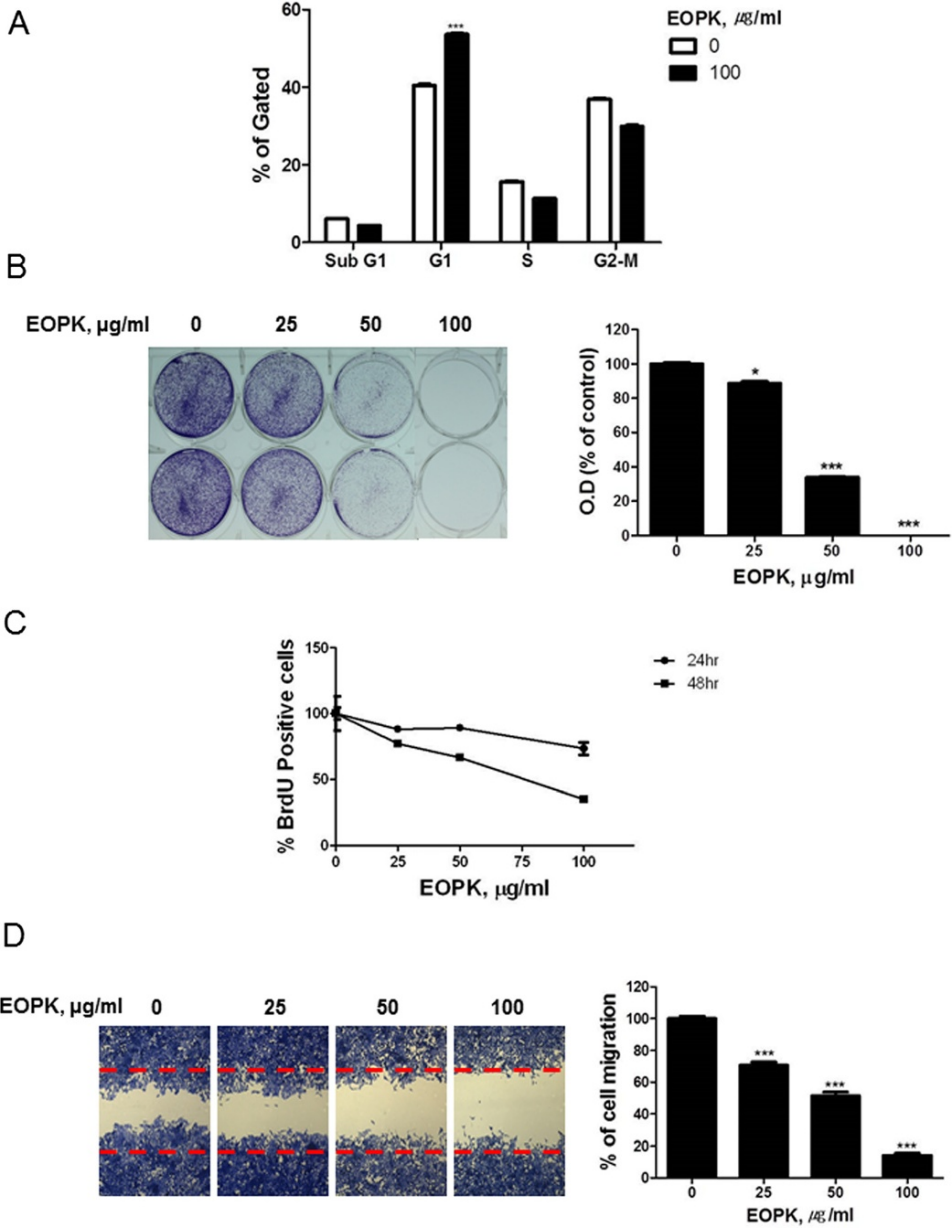
**EOPK inhibits cell proliferation and migration in HCT116 colorectal cancer cells. (A)** Cells were treated with EOPK (100 μg/ml) for 24 h. Cell cycle distribution was analyzed by flow cytometry. Bar graphs represent the percentage of sub-G1, G1, S, G2-M phase cells. Data represent mean ± SD of three independent experiments. *** *p* < 0.001 compared with untreated control. **(B)** HCT116 cells were treated with various concentrations of EOPK (25, 50, 100 μg/ml) and maintained for 5 days. The cells were resolved in 70% ethanol after washing with distilled water, and crystal violet absorbance was read using a microplate reader. Data represent mean ± SD of three independent experiments. * *p* < 0.05, ** *p* < 0.01 and *** *p* < 0.001 compared with control. **(C)** HCT116 cells were treated with EOPK for 24 h or 48 h and cell proliferation was measured by using a BrdU proliferation ELISA kit (Roche, Swiss). **(D)** Cells were treated with EOPK (25, 50, 100 μg/ml) for 24 h, and cell migration was assayed by wound healing assay. The number of cells migrating into the scratched area was photographed (×100) and calculated as a percentage of migration. Data are shown as the mean ± SD of three independent experiments. *** *p* < 0.001 compared with untreated control.

We next performed cell cycle analysis following EOPK treatment in HCT116 colorectal cancer cells. Cells were treated with 100 μg/ml of EOPK for 24 h, and were then analyzed by FACS. Treatment with EOPK significantly increased G1-arrest in HCT116 cells (Figure 2A).

To investigate whether EOPK suppressed the proliferation of human colorectal cancer cells, a cell growth assay was performed. This assay assessed the effect of EOPK on the long-term (5 d) growth of colorectal cancer cells. As shown in Figure 2B, EOPK significantly suppressed cell growth in a dose-dependent manner. Importantly, 100 μg/mL EOPK suppressed HCT116 growth 99.9%. Consistently, a BrdU assay showed that EOPK treatment inhibited the proliferation of HCT116 cells in a concentration- and time-dependent manner (Figure 2C).

To better understand the inhibitory effect of EOPK on cell migration, we used a wound-healing assay. As shown in Figure 2D, EOPK significantly decreased serum-induced cell migration by 29.4%, 48.6% and 85.8% as compared to the untreated control at 25, 50, and 100 μg/ml EOPK, respectively.

### EOPK attenuates β-catenin expression and AKT, and ERK phosphorylation via PAK1

**Figure 3:**
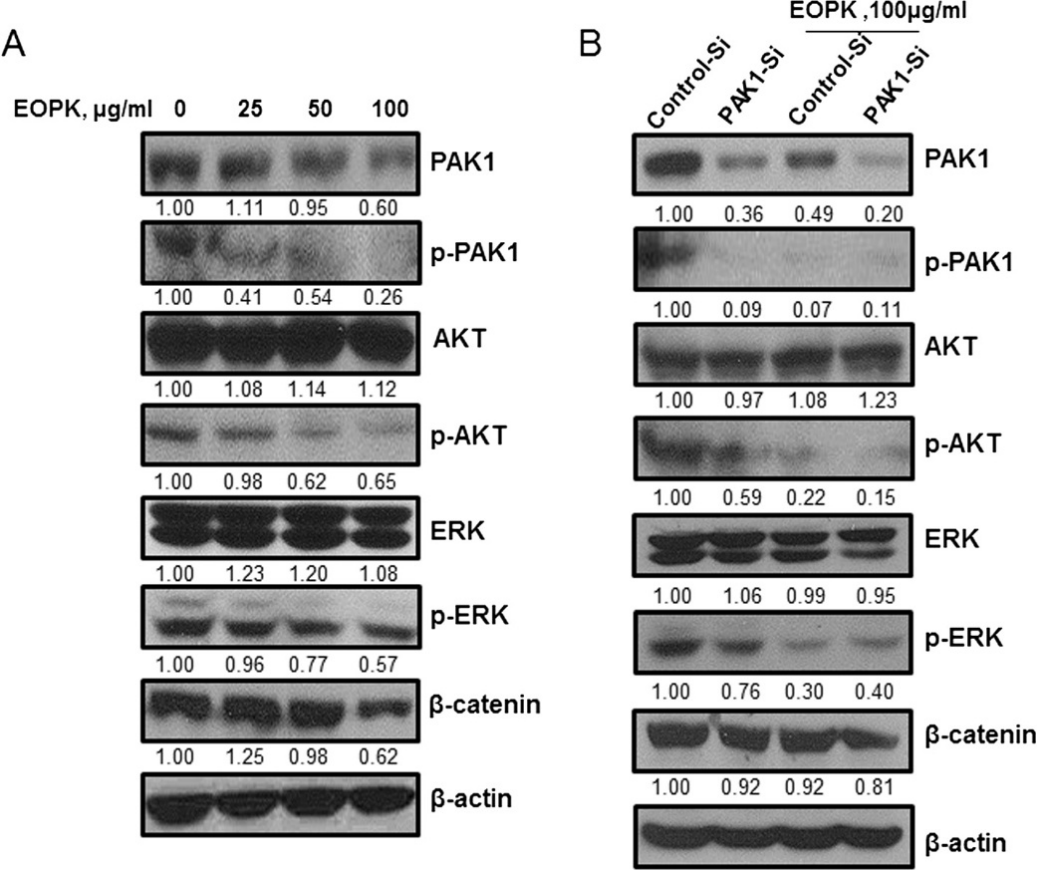
**EOPK suppresses AKT and ERK phosphorylation and β-catenin expression via PAK1 inhibition in HCT116 colorectal cancer cells. (A)** HCT116 cells were treated with various concentrations of EOPK (25, 50, 100 μg/ml). **(B)** HCT116 cells were transfected with PAK1-siRNA or a control siRNA for 48 h and incubated in the presence or absence of EOPK (100 μg/ml) for 24 h. For both experiments, cell lysates were prepared and subjected to Western blotting to determine the expression of PAK1, p-PAK1, AKT, p-AKT, ERK, p-ERK, β-catenin and β-actin. Band density of PAK1, p-PAK1, AKT, p-AKT, ERK, p-ERK, and β-catenin was quantified using Gelpro analyzer (Media Cybernetics, Bethesda, MD, USA).

To assess whether the PI3K/AKT, MAPK/ERK and Wnt/β-catenin signaling pathways contributed to the inhibitory effect of EOPK, the protein expression levels of PAK1, β-catenin, phosphor-AKT, and phosphor-ERK were investigated by Western blotting. EOPK remarkably suppressed the phosphorylation of AKT, ERK, and PAK1, as well as the expression of β-catenin in HCT116 colorectal cancer cells (Figure 3A). We previously showed that siRNA knockdown of PAK1 reduced phosphorylation AKT and ERK (Figure 1B). This is consistent with previous reports, in which, knockdown of PAK1 in colorectal cancer cells by shRNA suppressed cell proliferation via the ERK and AKT pathways [24, 25]. Interestingly, PAK1 knockdown enhanced the inhibitory effect of EOPK on β-catenin expression and phosphorylation of AKT and ERK in HCT116 cells (Figure 3B).

### PAK1 mediates EOPK-induced G1 arrest, suppression of cell proliferation and migration, and altered actin cytoskeletal arrangement

**Figure 4:**
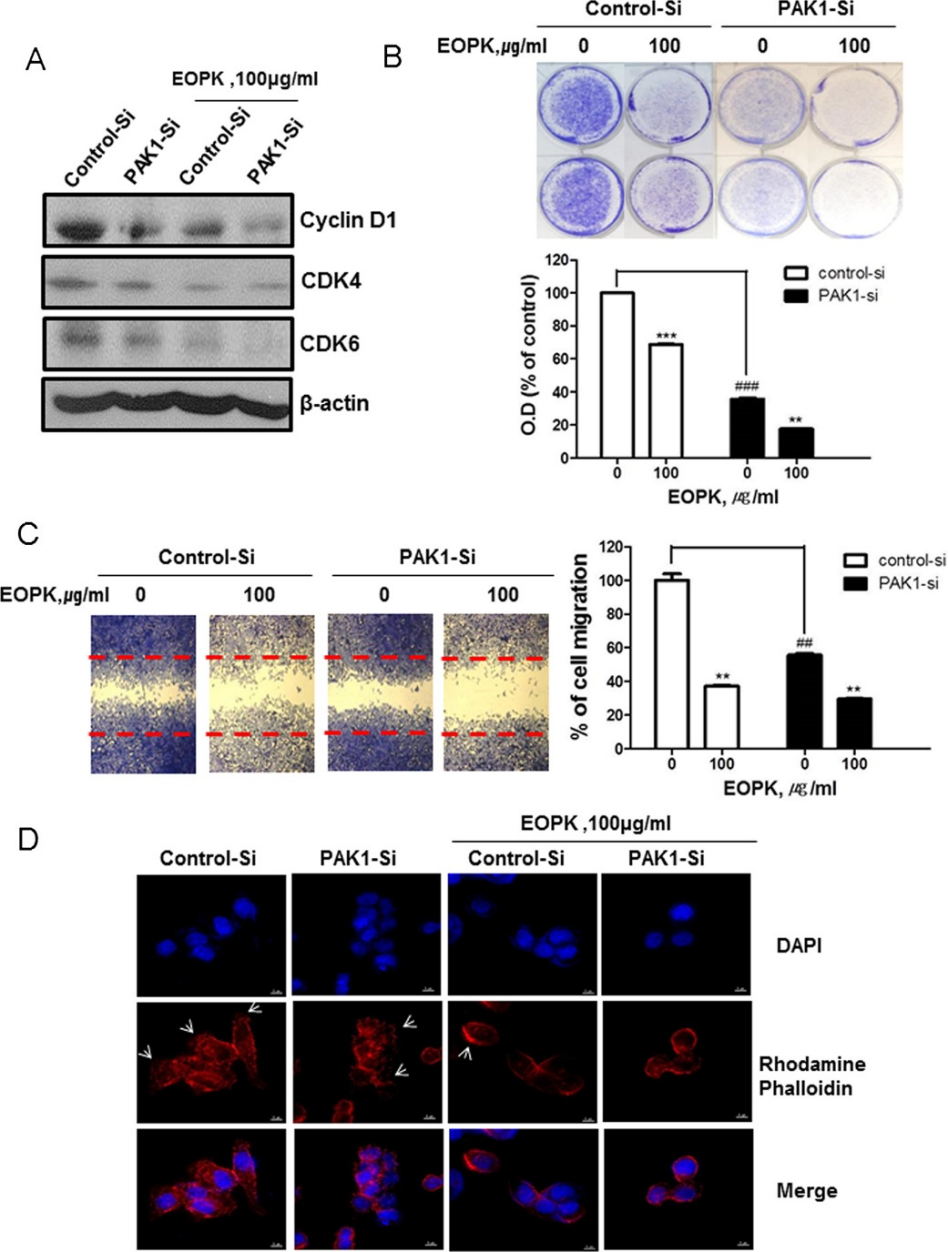
**PAK1 siRNA enhances the inhibitory effect of EOPK on the cell proliferation and migration. (A)** HCT116 cells were transfected with PAK1-siRNA for 48 h and were incubated in the presence or absence of EOPK (100 μg/ml) for 24 h. Cell lysates were prepared and subjected to Western blotting to determine the expression of Cyclin D1, CDK4, CDK6 and β-actin. **(B)** HCT116 cells were transfected with PAK1-siRNA for 24 h and treated with EOPK (100 μg/ml) for 5 days. The cells were resolved in 70% ethanol after washing with distilled water, and crystal violet absorbance was read using a microplate reader. Data represent mean ± SD of three independent experiments. * *p* < 0.05, ** *p* < 0.01 and *** *p* < 0.001 compared with control. ### *p* < 0.001 compared with control-siRNA and PAK1-siRNA. **(C)** Cell migration was assessed by a wound healing assay. The number of cells migrated into the scratched area was calculated. Data are shown as the mean ± SD of three independent experiments. *** *p* < 0.001 compared with untreated control. ### *p* < 0.001 compared with control-siRNA and PAK1-siRNA. **(D)** Fixed cells were stained with rhodamine phalloidin and DAPI, and imaged using a DV Elite™ Imaging System (Deltavision, Applied Precision, Inc., USA). Arrow indicates lamellipodia. Arrow indicates lamellipodia.

We next assessed whether EOPK-mediated G1 arrest is dependent on PAK1 inhibition. Knockdown of PAK1 by siRNA reduced the expression of G1 regulatory proteins, such as cyclin D1, CDK4, and CDK6 (Figure 4A). Consequently, PAK1 knockdown enhanced EOPK-mediated inhibition of proliferation, as assessed by cell growth assay (Figure 4B). Further, PAK1 knockdown also enhanced EOPK-mediated cell migration, as determined using a wound healing assay (Figure 4C). PAK1 plays a critical role in migration, invasion, and proliferation through its regulation of actin cytoskeletal reorganization [26, 27]. To determine whether the EOPK-mediated suppression of migration was due to inhibition of PAK1-mediated actin cytoskeletal rearrangement, an immunofluorescence assay was performed. In tis assay, rhodamine phalloidin was used to detect F-actin. Knockdown of PAK1 by siRNA reduced basal spread of cells and increased cell rounding. In addition, knockdown of PAK1 enhanced EOPK-mediated alterations of actin cytoskeleton in HCT116 colorectal cancer cells (Figure 4D).

## Discussion

Our findings show that EOPK efficaciously inhibits the proliferation and migration of colon cancer cells *in vitro.* Fluorouracil (5-FU) is a standard therapeutic agent used in the treatment of colorectal cancer since the 1950s. However, not all of patients respond to 5-FU [28]. Thus, recent work identifying the antitumor effects of natural products indicates they may be a promising alternative therapeutic for the treatment of colorectal cancer. Many extracts from vegetables, fruits, and plants, including epigallocatechin gallate (EGCG) and *Morus alba* leaf extract, can inhibit cell proliferation and survival, and induce apoptosis in colorectal cancer cell [29, 30]. Here, we show that EOPK significantly decreases proliferation and migration in colorectal cancer cells, without affecting normal cells. Recent reports have indicated that EOPK has anti-obesity and hypolipidemic effects [15, 16]. Moreover, essential oil from *P. koraiensis* seeds, leaves, nuts, and cones have been shown to have biological efficacy [17, 18, 20, 31, 32]. However, the effect of EOPK on tumor proliferation and survival has not been examined to date.

PAK1 was recently shown to be an effective therapeutic target for cancer treatment [33]. Interestingly, we found that EOPK treatment significantly reduced PAK1 expression in several colon cancer cells. It is well known that PAK1 mediates cell proliferation, migration, invasion, and survival through the ERK and AKT signaling pathways [6]. Thus, to confirm the involvement of PAK1 in EOPK-mediated inhibition of cell proliferation and migration, we analyzed the effects of EOPK on AKT and ERK phosphorylation in HCT116 cells. Knockdown of PAK1 with siRNA enhanced the inhibitory effect of EOPK on AKT and ERK phosphorylation in HCT116 cells. Further, PAK1 knockdown enhanced EOPK-mediated inhibition of PAK1 activity. Combined, these data suggest that EOPK suppresses cell growth and migration via the inhibition of PAK1, AKT, and ERK signaling in colon cancer cells. In addition, PAK1 is known to be involved in macrophage spreading and migration, and previous work indicates that PAK1 acts via ERK1/2 to regulate lamellipodia formation [34]. We showed that EOPK significantly suppressed actin cytoskeletal rearrangement in HCT116 colorectal cancer cells. Furthermore, EOPK significantly decreased proliferation and migration, induced G1-arrest, and inhibited the expression of phospho-ERK, phospho-AKT, β-catenin, cyclin D1 and CDK4/6 in colorectal cancer cells.

## Conclusions

In summary, our results demonstrate that EOPK reduces proliferation of HCT116 cells through G1 arrest, without affecting normal cells. EOPK inhibits cell migration and alters cytoskeletal structure, through the reduction of basal spread and cell elongation and increased cell rounding. EOPK-induced G1 arrest, inhibited cell proliferation and migration, and suppressed actin cytoskeletal rearrangement via the inhibition of PAK1. This inhibition of PAK1 resulted in decreases in ERK and AKT phosphorylation and β-catenin expression. Combined, our findings provide evidence that EOPK may be a novel therapeutic for the treatment of colorectal cancer. There is great potential for the development of novel chemotherapeutics from the leaves of *P. koraiensis*.

## Abbreviations

EOPK: Essential oils of *Pinus koraiensis* leaves
PAK1: p21-activated kinase 1.
